# Therapeutic Notes

**Published:** 1914-06

**Authors:** J. M. Fortescue-Brickdale


### Therapeutic Notes.

### CELL PROLIFERANTS.

Since 1908 several substances have been employed to promote the growth of epithelium on granulating surfaces and open wounds. They are all closely related chemically, and have many properties in common. They are insoluble in water, but more or less soluble in oils and fats, and are usually employed in 8 per cent. dilutions in oil, lanoline or vaseline. They are all red in colour and some are true dye stuffs. The simplest chemically is amido-azo-toluol :—

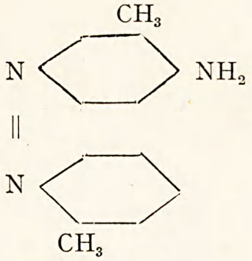

It is said to have a more energetic action than the original scarlet red.

The amido group ($NH_2$) contains two hydrogens capable of replacement by other radicles ; thus if acetyl ($CH_3CO$) replaces one hydrogen, a light yellowish red substance is produced, to which the trade name of *Azodermin* has been given. It is not so soluble in oils as scarlet red or amido-azo-toluol, and is only with difficulty dissolved in ether. When both the amido hydrogen atoms are replaced by acetyl a similar body is produced, which not being a true dye does not produce permanent stains on the skin or linen. It is known by the trade name of *Pellidol.* It is very soluble in oils and fats, and thus ointments prepared from it have none of the grittiness which is found with scarlet red. *Azodolen* is a mixture of equal parts of Pellidol and an organic iodine compound. Pellidol being very soluble may be employed in lower dilutions, such as 2 per. cent. The addition of the iodine compound is thought to render preliminary antiseptic applications unnecessary.

Scarlet red itself is the most complicated chemically of the series, as here the two hydrogens are replaced by a group called azo-$\beta$-naphthol, $NC_{10}H_6OH$. The solutions in oil are partly suspensions, which prevents them from being quite smooth in consistence. It may be conveniently prepared according to Bruhn's formula, which consists of 5 to 10 per cent. of the drug in equal parts of lanoline and paraffin ointment. These preparations being all more or less irritant, are best used for one or two days only, and then replaced for a similar period by boracic ointment. A careful cleaning of the surface with iodine or peroxide of hydrogen is also necessary, as they do not act well on discharging or septic wounds.

Injections of scarlet red dissolved in oil have been found to stimulate the growth of carcinoma in mice; the theory of its action appears to be that it is really cytotoxic,and that it is the products of cell disintegration which stimulate other cells to proliferate. In rabbits injections into the site of severe X-ray burns have caused necrosis and sloughing, but in slighter burns epithelial proliferation has resulted.

Two cases of poisoning have been reported which were distinctly alarming though not fatal. The symptoms were dizziness, pain in the stomach, nausea and vomiting.

Another substance, belonging to a different chemical group, which has been credited with similar properties is Allantoin:—

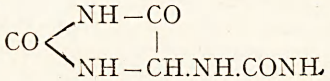

It is thought to be connected in some way with growth and cell proliferation both in plants and animals; in man it is apparently passed through the body unchanged, and the small amounts recovered from normal urine are probably derived from food. In some animals it appears to be a normal metabolic product.

It is certainly remarkable that both this substance and the scarlet red series should contain somewhat similar nitrogen groupings. Azodermin and Pellidol also contain CO groups. Allantoin is used in 0.3 to 0.4 watery solution as a dressing for ulcers and wounds. It occurs in the plant called comfrey, which has an ancient reputation as a healing application.

J. M. Fortescue-Brickdale.

